# The gene *YEF3* function encoding translation elongation factor eEF3 is partially conserved across fungi

**DOI:** 10.3389/fmicb.2024.1438900

**Published:** 2024-08-23

**Authors:** Giovanna Maldonado, Alejandra García, Saturnino Herrero, Irene Castaño, Michael Altmann, Reinhard Fischer, Greco Hernández

**Affiliations:** ^1^Laboratory of mRNA and Cancer, Unit of Biomedical Research on Cancer, National Institute of Cancer (Instituto Nacional de Cancerología, INCan), Mexico City, Mexico; ^2^Abteilung Mikrobiologie, Institut für Angewandte Biowissenschaften, Karlsruhe, Germany; ^3^División de Biología Molecular, Instituto Potosino de Investigación Científica y Tecnológica A.C (IPICYT), San Luis Potosí, Mexico; ^4^Institut für Biochemie und Molekulare Medizin (IBMM), Universität Bern, Bern, Switzerland; ^5^Escuela de Medicina y Ciencias de la Salud, Tecnológico de Monterrey, Mexico City, Mexico

**Keywords:** *YEF3*, *eEF3*, translation elongation, translation evolution, fungal translation, RNA metabolism

## Abstract

**Introduction:**

Translation is a fundamental process of life. In eukaryotes, the elongation step of translation is highly conserved and is driven by eukaryotic translation elongation factors (eEF)1A and eEF2. A significant variation of the elongation is the activity of eukaryotic elongation factor (eEF) 3 in *Saccharomyces cerevisiae* encoded by the gene yeast elongation factor (*YEF3*) with orthologs in all fungal species, a few algae, and some protists. In *S. cerevisiae, YEF3* is an essential gene and eEF3 plays a critical role in translation elongation, as it promotes binding of the ternary complex acylated-Transfer RNA (tRNA)—eEF1A—Guanosine-5'-triphosphate (GTP) to the aminoacyl (A) site of the ribosome, the release of uncharged tRNAs after peptide translocation, and ribosome recycling. Even though *YEF3* was discovered more than 40 years ago, eEF3 has been characterized almost exclusively in *S. cerevisiae*.

**Methods:**

We undertook an *in vivo* genetic approach to assess the functional conservation of *eEF3* across phylogenetically distant fungal species.

**Results:**

We found that *eEF3* from *Zygosaccharomyces rouxii* and *Candida glabrata* (both belonging to *phylum* Ascomycota), *Ustilago maydis* (*phylum* Basidiomycota), and *Gonapodya prolifera* (*phylum* Monoblepharomycota), but not *Aspergillus nidulans* (*phylum* Ascomycota), supported the growth of *S. cerevisiae* lacking the endogenous *YEF3* gene. We also proved that *eEF3* is an essential gene in the ascomycetes *C. glabrata* and *A. nidulans*.

**Discussion:**

Given that most existing knowledge on fungal translation has only been obtained from *S. cerevisiae*, our findings beyond this organism showed variability in the elongation process in Fungi. We also proved that *eEF3* is essential in pathogenic fungi, opening the possibility of using eEF3 as a target to fight candidiasis.

## Introduction

Translation plays a central role in gene expression in all forms of life (Hershey et al., 2019). The elongation step of translation consists of the decoding of a messenger ribonucleic acid's [[m]RNA] genetic information into protein by the ribosome and translation factors. In eukaryotes, this step requires the action of eukaryotic translation elongation factors (eEF) eEF1A and eEF2, as well as the nucleotide exchanger eEF1B (Dever et al., 2018). Elongation starts when an acylated-tRNA (also known as aminoacyl-tRNA, aa-tRNA) is brought to the ribosomal A site as part of the ternary complex aa-tRNA—eEF1A—GTP. Conformational changes in the ribosomal decoding center drive codon-anticodon pairing between aa-tRNAs and mRNA codons. Formation of the peptide bond is carried out at the ribosome peptidyl transferase center (PTC), between the incoming amino acid of the acylated-tRNA and the one positioned in the peptidyl (P) site (peptidyl-tRNA). Afterward, a peptide bond is formed, and eEF2 drives tRNA translocation from the A-P site to the P-E site so that the deacylated-tRNA is positioned at the exit (E) site and the peptidyl-tRNA is in the P site. GTP hydrolysis triggers eEF1A—GDP release, the uncharged tRNA leaves the exit (E) site, and the ribosome moves three nucleotides in the 5′-3′ direction to position the next codon at the empty A site. eEF1A is then recycled to its active form (eEF1A—GTP) by eEF1B. This cycle is repeated until an mRNA stop codon is placed at the free A site, which triggers peptide release from the ribosome to terminate translation. The ribosomal subunits dissociate, release the mRNA, and may enter into a new round of initiation (Choi et al., 2018; Dever et al., 2018).

A significant variation in translation elongation in eukaryotes is the requirement of eukaryotic elongation factor 3 (eEF3) in the budding yeast *S. cerevisiae*. During the elongation cycle, *eEF3* facilitates (1) the release of deacylated-tRNAs from the ribosomal E site after peptide translocation (Ranjan et al., 2021); (2) binding of the ternary complex to the A site (Triana-Alonso et al., 1995; Andersen et al., 2006); and (3) plays a role in the recycling process, as it promotes the disassembly of post-termination complexes into their components (Kurata et al., 2010). *YEF3*, which encodes eEF3, is an essential gene in *S. cerevisiae* (Qin et al., 1990). The orthologs of *YEF3*, termed *eEF3* genes, have been found *in silico* in all fungal species and some algae but not in animals, plants, or the vast majority of protists (Mateyak et al., 2016; Murina et al., 2019).

Despite its ubiquitous presence across the fungi kingdom and the fact that 48 years have passed since its discovery (Skogerson and Wakatama, 1976), *YEF3* and *eEF3* have been genetically, biochemically, and structurally characterized only in the yeast *S. cerevisiae*. The function of *YEF3* orthologs in species other than *S. cerevisiae* has been scarcely studied, and it is not known whether the *eEF3* gene is essential in different fungal species. In this study, we demonstrate that the function of *YEF3* is partially conserved across phylogenetically distant fungi. We also show that *eEF3* is an essential gene in *Candida glabrata* and *Aspergillus nidulans*, fungal species with enormous medical and research relevance.

## Materials and methods

### Databases search

We used 1,044 amino acids of the *S. cerevisiae* eEF3 protein to perform Basic local alignment search tool (BLAST) searches at: http://blast.ncbi.nlm.nih.gov/ and www.uniprot.org/blast/. eEF3 from the species selected for this study is listed in Table 1.

**Table 1:** *eEF3* from the fungal species selected for this study.

**Phylum^a^**	**Species**	***eEF3* ortholog NCBI^b^ ID**
Ascomycota	*Saccharomyces cerevisiae*	NP_013350.1
	*Zygosaccharomyces rouxii*	XM_002499110.1
	*Candida glabrata*	XM_445123.1
	*Aspergillus nidulans*	BN001301.1
		2841053.2843806
Basidiomycota	*Ustilago maydis*	XM_755206.1
Monoblepharomycota	*Gonapodya prolifera*	KXS20890.1

^a^Classification according to Wijayawardene et al. (2020).

^b^National Center for Biotechnology Information, USA.

### Genomic DNA extraction and plasmid construction

*eEF3 DNA* from *Gonapodya prolifera* and *A. nidulans* was *in vitro* synthesized with the preferential codon usage of *S. cerevisiae* into the pUC57-simple and pESC-TRP vectors, respectively, and purchased from GeneScript (Supplementary Table 1). *Z. rouxii* genomic DNA was purchased from the American Type Culture Collection (ATCC). Genomic DNA isolation from *S. cerevisiae, C. glabrata*, and *Ustilago maydis* was carried out using the Wizard Genomic DNA Purification Kit. The cloning for plasmid construction was achieved according to standard techniques (Sambrook and Russel, 1999).

An in-frame human influenza hemagglutinin (HA) protein tag sequence was introduced at the 3'-end of all *eEF3* DNAs. Polymerase chain reaction (PCR)-amplified DNA bands were cloned into the pTZ57R/T vector to create the respective pTZ57-*eEF3-HA* constructs (Supplementary Table 2). Clones without artifactual mutations were further subcloned onto the p301-*TRP1* vector to obtain the corresponding p301-*eEF3-HA* constructs. Recoded *eEF3-HA* DNAs (GeneScript) were subcloned from pUC57-simple onto the p301-*TRP1* vector.

For pVT-U-*YEF3* construction, *S. cerevisiae YEF3* was PCR-amplified using genomic DNA as a template and cloned into the vector pVT-U (Vernet et al., 1987). To construct the plasmid pYC12-*eEF3, C. glabrata eEF3* was PCR-amplified using genomic DNA as a template and cloned onto the vector pTZ57R/T. A DNA clone without artifactual mutations was further subcloned onto vector pYC12 (Yáñez-Carrillo et al., 2015). The *C. glabrata eEF3* KO-cassette *eEF3*Δ*::NATMX* was created consisting of 1,000 nucleotides upstream (genomic left border, LB) of the *eEF3* AUG start codon followed by the Nourseothricin resistance gene *(NATMX)* DNA and 1,000 nucleotides downstream of the *eEF3* gene stop codon (genomic right border, RB; Supplementary Table 4). The *eEF3* KO-cassette was further cloned into pTZ57R/T to create the plasmid pTZ57-cassette. The *NATMX* gene DNA was subcloned from the vector pAG25 (Goldstein and McCusker, 1999), and the different fragments of the *C. glabrata eEF3* gene were PCR-amplified using genomic DNA and cloned onto the pTZ57R/T. All constructs were corroborated by Sanger sequencing. Plasmids are listed in Table 2.

**Table 2:** Plasmids used in this study.

**Plasmid**	**Description**	**Source**
pTZ57 R/T	A vector for PCR fragment cloning possesses A-overhanging ends.	InsTAclone PCR Cloning Kit (ThermoFisher)
pUC57-simple	A vector to propagate any DNA fragment.	GeneScript
p301-*TRP1*	Contains the *TRP1* gene as an auxotrophic marker and the inducible *Gal10* promoter for controlled expression in *S. cerevisiae*.	Hans Trachsel, Bern University
pVT-U	Contains the *URA3* gene as an auxotrophic marker and the *Alcohol dehydrogenase (ADH)* promoter for constitutive expression in *S. cerevisiae*.	Vernet et al., 1987
pYC12	Contains the gene *URA3* as an auxotrophic marker and the promoter of *TEF1 for* constitutive expression in *C. glabrata*.	Yáñez-Carrillo et al., 2015
pAG25-NATMX	Contains the *NATMX* gene DNA that confers resistance to the antibiotic cloNAT.	Addgene, plasmid ID #35121
pVT-U-*YEF3*	Contains the *S. cerevisiae YEF3* DNA that was cloned onto pVT-U.	This study
pTZ57 *eEF3 glabrata*	Contains the *C. glabrata eEF3* DNA on pTZ57 R/T.	This study
pTZ57 *eEF3* KO-cassette	Contains the *C. glabrata eEF3* KO-cassette *eEF3Δ::NATMX*	This study
pYC12-*eEF3*	*C. glabrata eEF3* DNA was cloned onto pYC12.	This study
pUC57-*eEF3-HA prolifera*	*G. prolifera eEF3-HA* DNA was cloned onto pUC57-simple.	This study
p301-*YEF3-HA cerevisiae*	*S. cerevisiae eEF3-HA* DNA was cloned onto p301-*TRP1*.	This study
p301-*eEF3-HA glabrata*	*C. glabrata eEF3-HA* DNA was cloned onto p301-*TRP1*.	This study
p301-*eEF3*-HA *rouxii*	*Z. rouxii eEF3-HA* DNA was cloned onto p301-*TRP1*.	This study
p301-*eEF3-HA maydis*	*U. maydis eEF3-HA* DNA was cloned onto p301-*TRP1*.	This study
pESC-TRP-*eEF3-HA nidulans*	*A. nidulans eEF3-HA* DNA cloned onto pESC-*TRP1*.	This study
p301-*eEF3-HA prolifera*	*G. prolifera eEF3-HA* DNA cloned onto p301-*TRP1*	This study
pMCB17apx	pMCB17 version for fusion of Green fluorescent protein (GFP) to N-termini of proteins of interest in *Aspergillus nidulans*.	Efimov et al., 2006
pSH114	*A. nidulans eEF3* genomic sequence from ATG to stop in pMCB17apx.	This study
pFN03	It expresses the *A. fumigatus pyrG* marker.	Aysha H. Osmani, Ohio State University

### Phenotypic rescue of the *YEF3* deletion in *S. cerevisiae*

Protocols for yeast growth and gene deletion were according to Nosek and Tomaska (2013) and Xiao (2014). All strains used in this study are listed in Table 3.

**Table 3:** Strains used in this study.

**Species**	**Genotype**	**Source**
*S. cerevisiae*	*ade2, trp1, leu2, his3, ura3, yef3Δ::KANMX, < *pVT*-*U*-YEF3 S. cerevisiae>*	This study
*S. cerevisiae*	*ade2, trp1, leu2, his3, ura3, yef3Δ::KANMX, < *pVT*-*U*-YEF3>*, < p301-*TRP1*>	This study
*S. cerevisiae*	*ade2, trp1, leu2, his3, ura3, yef3Δ::KANMX, < *pVT*-*U*-YEF3>*, < p301-*TRP1-eEF3-HA S. cerevisiae*>	This study
*S. cerevisiae*	*ade2, trp1, leu2, his3, ura3, yef3Δ::KANMX, < *pVT*-*U*-YEF3>*, < p301-*TRP1-eEF3-HA C. glabrata*>	This study
*S. cerevisiae*	*ade2, trp1, leu2, his3, ura3, yef3Δ::KANMX, < *pVT*-*U*-YEF3>*, < p301-*TRP1-eEF3-HA Z. rouxii*>	This study
*S. cerevisiae*	*ade2, trp1, leu2, his3, ura3, yef3Δ::KANMX, < *pVT*-*U*-YEF3>*, < p301-*TRP1-eEF3-HA U. maydis*>	This study
*S. cerevisiae*	*ade2, trp1, leu2, his3, ura3, yef3Δ::KANMX, < *pVT*-*U*-YEF3>*, < pESC-*TRP1-eEF3-HA A. nidulans*>	This study
*S. cerevisiae*	*ade2, trp1, leu2, his3, ura3, yef3Δ::KANMX, < *pVT*-*U*-YEF3>*, < p301-*TRP1-eEF3-HA G. prolifera*>	This study
*C. glabrata*	*ura3Δ:::Tn903NeoR*	Cormack and Falkow, 1999
*C. glabrata*	*ura3Δ::Tn903NeoR < *pYC12>	This study
*C. glabrata*	*ura3Δ::Tn903NeoR, < *pYC12-*eEF3 C. glabrata*>	This study
*C. glabrata*	*ura3Δ::Tn903NeoR, eEF3Δ::NATMX, < *pYC12-*eEF3 C. glabrata*>	This study
*A. nidulans*	*wA3, pyrG89, pyroA4, veA1* (Strain GR5)	Waring et al., 1989
*A. nidulans*	*pyrG89, argB2, nkuAΔ*::*argB, pyroA4, veA1* (Strain TN02A3)	Nayak et al., 2006
*A. nidulans*	*wA3, pyrG89::eEF3Δ-Af-pyrG, pyroA4, veA1*	This study
	*eEF3 A. nidulans* KO-cassette in GR5 (heterokaryon), (Strain SSH182)	
*A. nidulans*	pSH114 in TN02A3, *alcA(p)::GFP::eEF3 A. nidulans* (full length) *pyrG89::Nc-pyr-4, argB2, nkuAΔ*::*argB, pyroA4, veA1* (Strain SSH181)	This study

Phenotypic rescue assays were performed according to Altmann et al. (1989), consisting of *S. cerevisiae* endogenous *YEF3* replacement by *eEF3* from other species. We used the haploid *S. cerevisiae* strains *ade2, trp1, leu2, his3*, and *ura3* to delete using homologous recombination the gene *YEF3* encoding for *eEF3* with the kanamycin resistance gene *(KANMX)* that confers resistance to the antibiotic kanamycin. The *YEF3* function is rescued by the *S. cerevisiae YEF3* DNA into the vector pVT-U (Vernet et al., 1987), giving rise to the strains *ade2, leu2, trp1, ura3, his3, yef3*Δ*::KANMX*, < pVT-U-*YEF3*>. This strain was transformed with the constructs p301-*eEF3-HA* or pESC-TRP-*eEF3*-HA expressing *eEF3* DNA from different species (Table 1) under the Gal10 promoter. Cells were grown in plates with minimal medium (yeast nitrogen base) (S) containing 2% glucose (D) supplemented with adenine (2 mg/mL), histidine (20 mg/mL), and leucine (10 mg/mL) and incubated at 30°C for 3 days. Subsequently, individual colonies were randomly selected and plated on S containing 2% galactose (to switch on the Gal10 promoter) and supplemented with adenine (2 mg/mL), histidine (20 mg/mL), and leucine (10 mg/mL). Cells were plated on the same medium supplemented with 5-Fluoroorotic acid hydrate (5-FOA; 1 g/L) and uracil (10 mg/mL) to trigger the removal of < pVT-U-*YEF3*> and further incubated at 30°C for 5 days.

### *eEF3* deletion in *C. glabrata*

To delete *C. glabrata eEF3, C. glabrata* strain *ura3*Δ*::Tn903NeoR* (Cormack and Falkow, 1999) was transformed with the pYC12-*eEF3* and plated onto a minimal medium (yeast nitrogen base + glucose, SD) supplemented with a dropout lacking uracil (casamino acids, CAA; Takara Bio Inc.) as an auxotrophic marker. The resulting yeast genotype was *ura3*Δ*::Tn903NeoR* < pYC12-*eEF3*>. To delete the *C. glabrata eEF3* gene, the *C. glabrata* strain *ura3*Δ*::Tn903NeoR*<*pYC12-eEF3*> was transformed with 600 ng of the *C. glabrata eEF3* KO-cassette (which contains NATMX gene that confers resistance to nouseothricin) and grown in Yeast extract peptone dextrose (YPD) medium supplemented with the antibiotic clonNAT (200 μg/mL) to select the *C. glabrata* strain *ura3*Δ*::Tn903NeoR, eEF3*Δ*::NATMX* < pYC12-*eEF3*>. Several isolated colonies growing in this medium were randomly selected, and their genomic DNA was isolated. The correct deletion of the *eEF3* gene by the cassette insertion was further corroborated by PCR. We obtained five colonies (numbered 4–8) of the desired genotype. Colonies were plated onto a minimal medium (SD + CAA) + uracil (growth control) or minimal medium + 5-FOA + uracil to get rid of < pYC12-*eEF3*>.

### *eEF3* deletion in *A. nidulans*

*A. nidulans* was cultured on supplemented minimal medium (MM) using standard procedures described by Hill and Käfer (2001). For the deletion of *eEF3*, we followed the heterokaryon-rescue method as described by Osmani et al. (2006). The flanking regions of *eEF3* Open reading frame (ORF) were amplified with specific primers (Supplementary Table 4). The genomic left border (LB) was amplified with primers *eEF3*-P1 and P3, and the genomic RB was amplified with primers P5 and P8. The *Aspergillus fumigatus pyrG* gene was used as an auxotrophic marker, and it was amplified from the plasmid pFN03 by using the primers pyrG-F and pyrG-R (Osmani et al., 2006). The three DNA fragments LB, RB, and *pyrG* were joined together with the PCR-fusion method using nested primers P2 and P7 (Szewczyk et al., 2006). The resulting PCR product was transformed as described previously in the *pyrG*^−^ auxotrophic strain GR5 (Waring et al., 1989). Transformants were screened by PCR for the homologous integration event.

### Microscopy

Bright field, differential interference contrast (DIC), and fluorescence images were taken at room temperature with an Axiophot Microscope (Zeiss), Germany. For GFP imaging, spores were incubated overnight on cover slides in 0.5 ml of liquid MM supplemented with 2% glycerol (de-repression of the *alcA* promoter). Images were taken the next day with a 63X immersion objective. For imaging of the KO-transformants, spores were grown in liquid or solid (0.8% agarose) MM supplemented with uracil and uridine (+UU) or without uracil and uridine (–UU). Images with the bright field 10X objective were taken directly from Petri dishes. Images were collected and analyzed with the Zen Software (Zeiss), Germany.

### Immunoblotting

Yeast cells were grown to O.D. = 1, pelleted, and further lysed on a frozen mortar in 100 mM KCl, 20 mM HEPES-KOH pH 7.6, 0.2 mM EDTA, 10% Glicerol, 0.1% Tritón X-100, 7 mM β-Mercaptoetanol and an EDTA-free Proteases Inhibitor Cocktail (Roche), Swiss. Protein concentration was determined using the Bradford Reagent (BioRad), United States according to the manufacturer's instructions. A volume of 20 mg of total protein lysate was resolved on 8% SDS-polyacrylamide gels and either stained with Coomassie blue (loading control) or blotted onto nitrocellulose membranes. Membranes were proved with a monoclonal anti-HA-HRP antibody (Santa Cruz, sc-7392). The expected size of proteins is shown in Supplementary Table 3.

## Results

### *eEF3* from phylogenetically distant species cross-complements *YEF3* in *S. cerevisiae*

We analyzed whether the function of *eEF3* is conserved in distant fungal species. We retrieved *YEF3* ortholog sequences from the different databases and six species were selected from across four *phyla*, namely *Z. rouxii, Candida glabrata*, and *A. nidulans* (all three belonging to the *phylum* Ascomycota), *U. maydis* (belonging to the *phylum* Basidiomycota), and *G. prolifera* (belonging to the *phylum* Monoblepharomycota; Wijayawardene et al., 2020).

**Figure 1:**
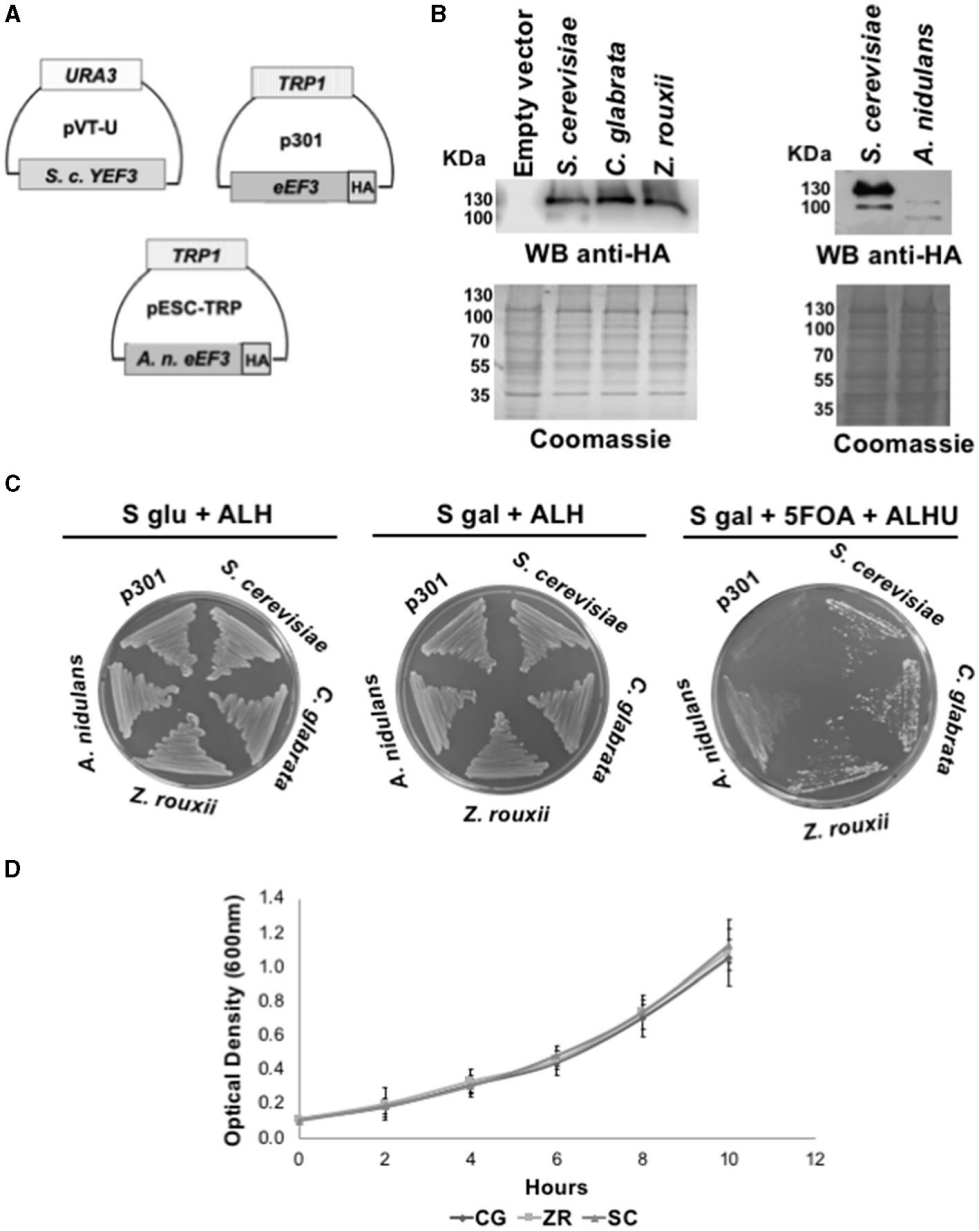
*eEF3* from different *Ascomycota* species differentially complements *S. cerevisiae YEF3*. **(A)** The scheme of plasmids pVT-U-*YEF3* containing the *S. cerevisiae YEF3* (*left*), p301*-eEF3-HA* HA-tagged versions of *eEF3* DNAs from different species used to perform the phenotypic rescues **(right)**, and pSEC-TRP containing the *A. nidulans eEF3-HA*. **(B)** Western blotting analysis using an anti-HA antibody (up) and Coomassie blue stain of total protein extracted from different species (down). **(C)** Phenotypic rescue using *eEF3* from *Ascomycota* species. Experiment controls: Empty vector (*p301*, negative control), *S. cerevisiae* (positive control). *eEF3* DNAs from *C. glabrata, Z. rouxii*, and *A. nidulans* were tested. The media composition is shown. Plates *S glu* + *ALH* (p301-*eEF3-HA* or pSEC-TRP-*eEF3-HA* expression is off) and *S gal* + *ALH* (p301-*eEF3-HA* or pSEC-TRP-*eEF3-HA* expression is on) are growth controls. Plates *S gal* + *ALHU* + *5-FOA* have expelled pVT-U-*YEF3*, and the growth relies only on p301-*eEF3-HA* or pSEC-TRP-*eEF3-HA* (*Phenotypic rescue*). **(D)** Growth curves of yeast cells expressing *eEF3* from *S. cerevisiae, C. glabrata*, or *Z. rouxii*. *CG, Candida glabrata; ZR, Z. rouxii; SC, Saccharomyces cerevisiae; A, Adenine; L, Leucine; H, Histidine; U, Uracil; 5-FOA*, 5-fluoroorotic acid hydrate.

Through homologous recombination and plasmid shuffling experiments, we performed phenotypic rescue assays (Altmann et al., 1989), consisting of heterologous complementation of the *S. cerevisiae YEF3* plasmid with the *eEF3* DNA from the species mentioned above. We generated the haploid *S. cerevisiae* strain Δ*yef3*::*KANMX*, < pVT-U-*YEF3*>, in which we deleted the *YEF3* gene with the *KANMX* gene and introduced the constitutively expressed *S. cerevisiae YEF3* DNA in the vector pVT-U (Figure 1A). This strain was afterward transformed with the *eEF3* DNA from the ascomycetes *C. glabrata* and *A. rouxii*, which contain an in-frame HA tag in the p301 vector (Figure 1A). In the case of *A. nidulans*, the *eEF3* DNA with an in-frame HA tag was cloned into the high-copy pESC-TRP vector (Figure 1A). Both vectors are under the inducible galactose (Gal10) promoter. To verify the *eEF3*-HA protein expression, we performed Western blotting analysis of total protein extracts using an anti-HA antibody (Figure 1B). Subsequently, S-gal plates were plated in S-gal medium supplemented with 5-FOA to expel the plasmid pVT-U-*YEF3*. Therefore, yeast growth depends solely on the expression of p301-*eEF3-HA* or pSEC-TRP-*eEF3-HA* in the case of *A. nidulans*. We observed that *eEF3-HA* DNA *from C. glabrata, Z. rouxii*, and *S. cerevisiae* (positive control), but not the empty vector, supported the growth of *S. cerevisiae* lacking the endogenous *YEF3* gene (Figure 1C). Although the *eEF3*-HA cDNA from *A. nidulans* was cloned in a high-copy vector, *eEF3*-HA was barely expressed compared to the other HA-*eEF3* (Figure 1B), and the low *eEF3*-HA synthesized was not able to support the growth of *S. cerevisiae* lacking the endogenous *YEF3* gene. The growth curves of the strains that rescued the function of *S. cerevisiae YEF3* showed similar growth patterns (Figure 1D).

**Figure 2:**
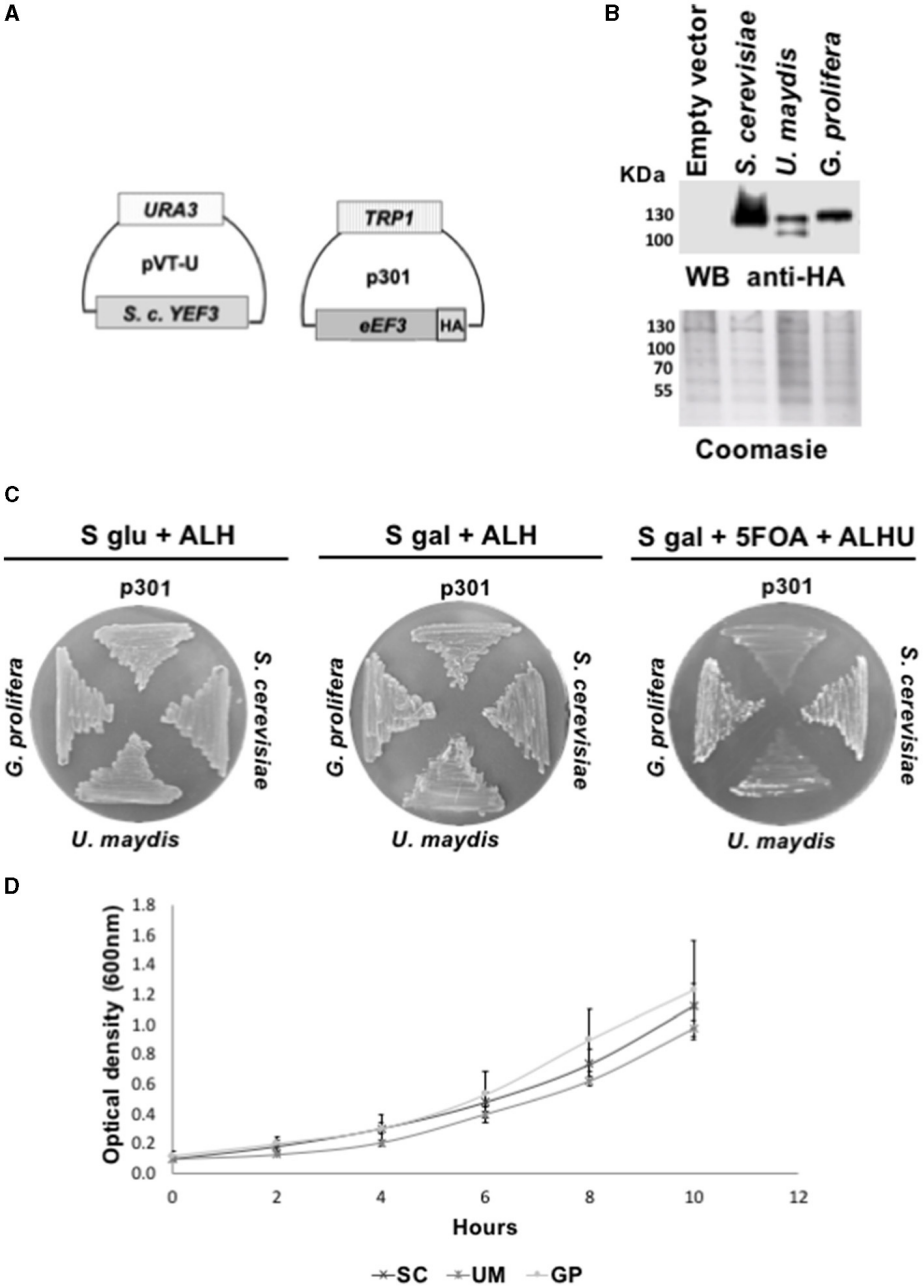
*eEF3* from different *phyla* species differentially complements *S. cerevisiae YEF3*. **(A)** Scheme of plasmids pVT-U-*YEF3* containing the *S. cerevisiae YEF3* (*left*) and p301*-eEF3-HA* containing HA-tagged versions of *eEF3* DNAs from different species used to perform the phenotypic rescues. **(B)** Western blotting analysis using an anti-HA antibody (up) and Coomassie blue stain of total protein extracted from different species (down). **(C)** Phenotypic rescue using *eEF3* from species belonging to non-*Ascomycota* fungi. Experiment controls: Empty vector (*p301*, negative control), *S. cerevisiae* (positive control). *eEF3* DNAs from the basidiomycete *U. maydis* and the monoblepharomycete *G. prolifera* were tested. The media composition is shown. Plates *S glu* + *ALH* (p301-*eEF3-HA* expression is off) and *S gal* + *ALH* (p301-*eEF3-HA* expression is on) are growth controls. Plates *S gal* + *ALHU* + *5-FOA* have expelled pVT-U-*YEF3*, and the growth relies only on p301-*eEF3-HA* (*Phenotypic rescue*). **(D)** Growth curves of yeast cells expressing *eEF3* from *S. cerevisia*e, *U. maydis*, or *G. prolifera*. *UM, Ustilago maydis; GP, Gonapodya prolifera; SC, Saccharomyces cerevisiae; A, Adenine; L, Leucine; H, Histidine; U, Uracil; 5-FOA*, 5-fluoroorotic acid hydrate.

We next analyzed *eEF3* from distant non-*Ascomycota phyla* using the same genetic approach and verified the expression of eEF3-HA proteins encoded by the plasmid p301-TRP1 (Figure 2A) by performing Western blotting experiments (Figure 2B). We observed that *U. maydis* and *G. prolifera eEF3-HA* also rescued the function of *S. cerevisiae YEF3* (Figure 2C). The growth curves were similar for all the organisms that did complement (Figure 2D).

### *eEF3* is an essential gene in *C. glabrata* and *A. nidulans*

Genetic experiments demonstrated that *YEF3* is an essential gene in *S. cerevisiae* (Qin et al., 1990). However, whether the *YEF3* orthologs are essential in other species has not been investigated. To address this question, we performed chromosomal recombination and plasmid shuffling experiments to delete *eEF3* with a KO-cassette with a selection marker. Its homologous integration disrupts the original gene, which is substituted by the resistance cassette.

**Figure 3:**
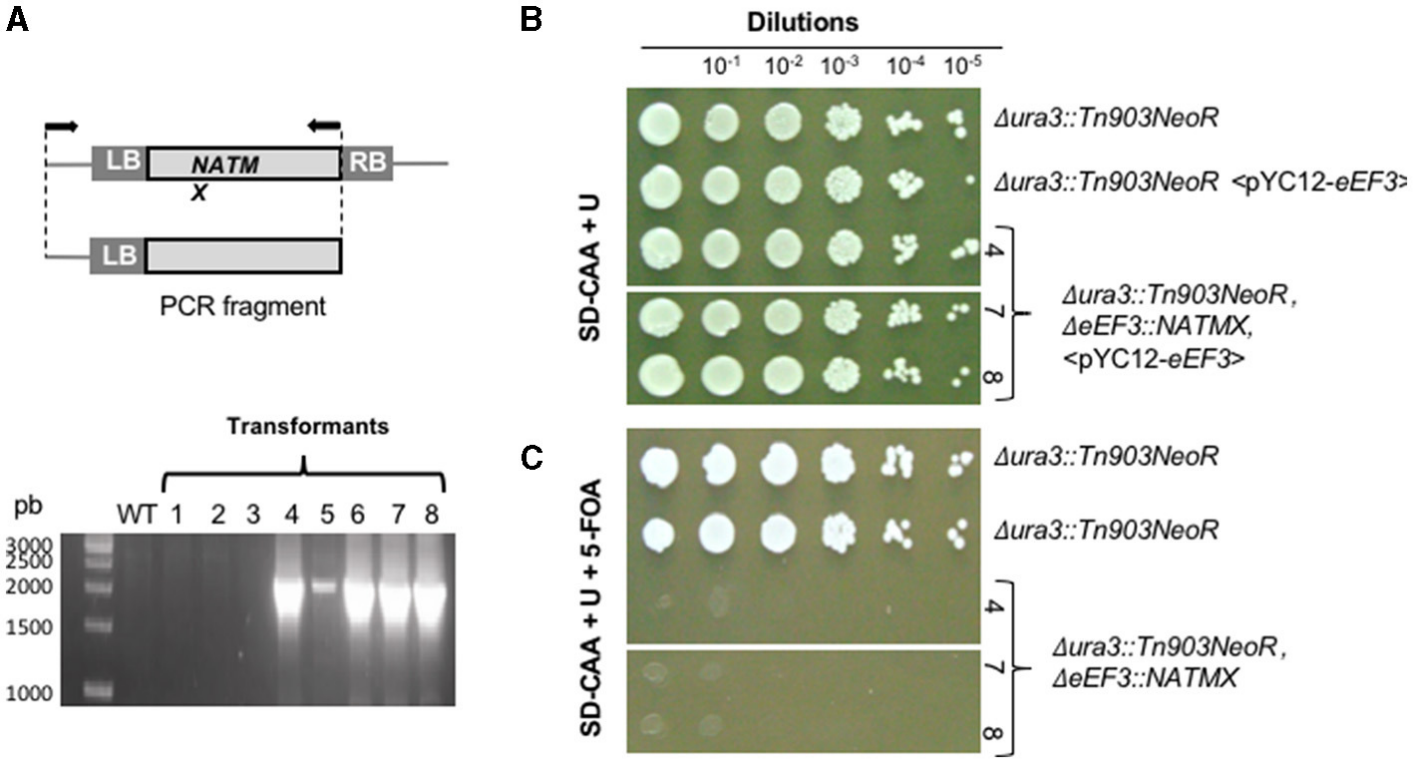
*eEF3* is an essential gene in *C. glabrata*. [**(A)**, upper] *eEF3* KO-cassette was used to delete the endogenous *eEF3* gene. *LB*, genomic left border; *RB*, genomic right border. A scheme of the amplified fragment of the *eEF3* KO-cassette integrated onto the *eEF3 locus* is depicted. Arrows indicate the position of the used primers. (*Lower*) PCR analysis of independent KO-transformants (lanes *1–8*). **(B, C)** KO-strains that integrated the *eEF3* KO-cassette. **(B)** Growth controls. **(C)** Lethality tests. The presence of 5-FOA triggers the expulsion of the pCY12-*eEF3* plasmid. The pYC12-*eEF3* loss leads to lethality phenotypes only in the strain Δ*ura3*::*Tn903NeoR*, Δ*eEF3*::*NATMX* lacking endogenous *eEF3* gene. The independent transformants *4, 7*, and *8* were tested, obtaining identical results. Genotypes in all cases are described. *SD-CAA*, minimal medium containing casamino acids; *U*, uracil; *5-FOA*, 5-fluoroorotic acid hydrate.

For *C. glabrata*, we constructed the *eEF3* KO-cassette *eEF3*Δ*::NATMX*, consisting of the *NATMX* marker that confers resistance to the antibiotic cloNAT, flanked by regions of the *eEF3* gene (Figure 3A, upper). Simultaneously, the *C. glabrata eEF3-HA* DNA in the vector pYC12, which constitutively expresses *eEF3*, was introduced in cells of the strain *ura3*Δ*::Tn903NeoR, eEF3*Δ*::NATMX* to generate the strain *ura3*Δ*::Tn903NeoR, eEF3*Δ*::NATMX*, < pYC12-*eEF3-HA*> in which we can control the lack of *eEF3*. To corroborate the correct homologous integration of the *eEF3 KO-*cassette, we amplified a fragment of DNA that spans from within *NATMX* to the adjacent region of this cassette. We found the integration of the cassette in several independent transformants, except in the wild-type cells (Figure 3A, lower).

We next performed a growth assay in the presence of 5-FOA that was selected for cells that had lost the pYC12-*eEF3* plasmid. Serial dilutions at a 1:10 ratio were plated onto MM supplemented with uracil (growth control; Figure 3B) and on MM containing uracil and 5-FOA (Figure 3C). Since endogenous *eEF3* is deleted and the plasmid pYC12-*eEF3* was expelled in the presence of 5-FOA, the absence of growth of *ura3*Δ*::Tn903NeoR, eEF3*Δ*::NATMX* cells in the latter medium indicates that *eEF3* is an essential gene in *C. glabrata* (Figure 3C). We obtained identical results in three independent colonies in which a successful deletion of endogenous *eEF3* was carried out.

We also assessed whether *eEF3* of *A. nidulans* is essential. To address this, we next performed the heterokaryon rescue technique (Osmani et al., 2006) that allows the production of polynucleated protoplasts with two genetically distinct types of nuclei, i.e., one possesses the deleted gene and the other contains the wild-type allele. For this aim, we constructed an *eEF3* KO-cassette that consisted of the *A. fumigatus pyrG*^+^ gene as an auxotrophic marker and flanking regions of the *eEF3* open reading frame. We transformed the *pyrG*^−−^ auxotrophic strain GR5 caused by the *pyrG89* mutation that requires UU to grow (Waring et al., 1989) with the *eEF3* KO-cassette that complements the UU auxotrophy. In this method, the homologous integration event at the *eEF3* locus occurs in only one of the nuclei, and a multinucleated heterokaryon strain is created containing both wild-type and *eEF3*Δ nuclei. Thus, the heterokaryon grows on selective minimal media lacking (–UU) and on non-selective minimal media (+UU). The conidia are mononucleated, and the GR5 grows on +UU but not on –UU minimal media. In contrast, conidia containing the *eEF3* KO-cassette can grow in selective –UU minimal media too. If the *eEF3* cassette is integrated at the *eEF3* locus and the deletion is lethal, the spores of the transformed conidia cannot grow in any medium.

**Figure 4:**
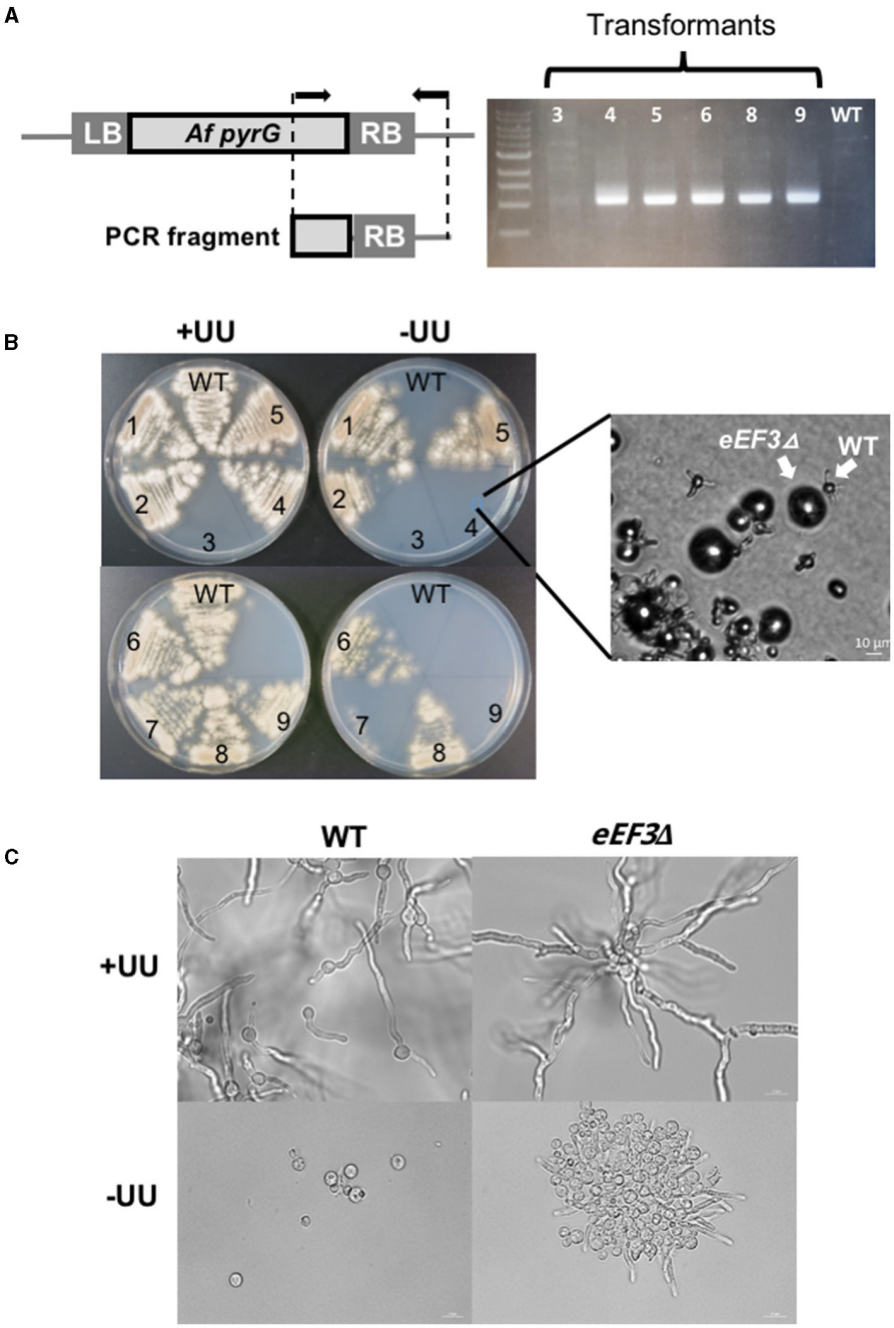
*eEF3* is an essential gene in *A. nidulans*. **(A)** PCR analysis of the KO-strains. A scheme of the amplified *A. fumigatus* (*Af*) fragment of the KO-cassette integrated onto the *eEF3 locus* is depicted. Arrows indicate the position of the used primers. *LB*, genomic left border; *RB*, genomic right border (RB). **(B)** Spores of the wild-type strain (GR5) and several transformants with the integrated *eEF3* KO-cassette were streaked out onto minimal medium (MM) plates with (+*UU*) or without (–*UU*) uracil and uridine. *Inset:* a microscopic picture of the spores taken directly from the Petri dish. **(C)** DIC microscopic pictures of the wild-type strain and transformant number 4. Spores were cultivated O/N on liquid MM with (+*UU*) or without (–*UU*) uracil and uridine at 28°C.

To verify the homologous integration of the selection marker, we amplified a fragment of DNA that spans from *pyrG* to the adjacent region of the *eEF3* KO-cassette (Figure 4A). We found homologous integration of the *eEF3* KO-cassette in all the tested transformants except transformant 3. As expected, no amplification was obtained in the wild-type cells (negative control). We observed that transformant numbers 4, 7, and 9 did grow on +UU but not on –UU (Figure 4B) media, indicating that only the wild-type conidia germinated on +UU. Spores were further cultivated overnight in liquid selective –UU or non-selective +UU minimal media. Microscopic analyses showed that the *eEF3*Δ spores germinated in selective minimal media –UU and produced up to three germination tubes, but they could not grow further (Figure 4B inset and Figure 4C). The growth of transformants 1, 2, 5, 6, and 8 can be explained by an additional heterologous integration event of the selection marker. We conclude that *eEF3* is an essential gene in *A. nidulans*.

## Discussion

In this study, we demonstrated that *eEF3* from the fungal species *Z. rouxii, C. glabrata, U. maydis*, and *G. prolifera* supports the growth of *S. cerevisiae* lacking endogenous *YEF3*. We could not determine whether or not *A. nidulans eEF3* complements the lack of *eEF3* in *S. cerevisiae*. The low expression level of *A. nidulans eEF3* that we systematically observed might be the result of DNA recombination, protein degradation, or both, as the protein might be harmful to *S. cerevisiae*. *eEF3* from other *Ascomycota* species, including *Schizosaccharomices pombe* (Mateyak et al., 2018) and *Candida albicans* (Di Domenico et al., 1992; Myers et al., 1992), and the basidiomycete yeast *Cryptococcus neoformans* (Blakely et al., 2001), also cross-complement *S. cerevisiae YEF3*. *eEF3* from a fungus-like species, namely the parasitic oomycete *Phytophthora infestans*, also cross-complements *S. cerevisiae YEF3* (Mateyak et al., 2018).

Recent *in silico* studies have discovered *eEF3* orthologs in various non-fungal lineages, such as green and red algae, choanoflagellates, heterokonts, dinoflagellates, cryptophytes, and oomycetes (Mateyak et al., 2016; Murina et al., 2019), and in viruses infecting the algae *Chlorella* (Yamada et al., 1993) and *Phaeocystis* (Murina et al., 2019). It would be interesting to investigate whether algal, protists, and viral *eEF3* orthologs are involved in translation.

### Rational design of fungicides: *eEF3* in the spotlight

The biotechnological and clinical importance of fungal infections has been dramatically growing. However, the control of fungal pathogens is currently limited due to the small number of effective fungicides and the lack of suitable targets (Kim et al., 2020). Here, we have shown that *eEF3* is an essential gene in *C. glabrata* and *A. nidulans*, two ascomycetes with strong medical and research relevance, respectively (Turner and Butler, 2014; Glöckner and Cornely, 2015; Park et al., 2017; Chen et al., 2021; Hernandez-Ramirez et al., 2021). This is the first study showing the essentiality of the *eEF3* gene beyond *S. cerevisae*. Since no plant or mammalian *eEF3* ortholog exists, our observations open for the first time the possibility of using rationally designed drugs to target *eEF3* to fight human fungal infections by these species, as well as other infections by harmful fungal species (Sturtevant, 2002). These drugs would be an alternative to the current antifungal drugs, which are based on azoles that disrupt cell walls but display inefficient results overall.

### ATP-binding cassette (ABC)-ATPases and the origin of *eEF3*

The evolutionary origin of *eEF3* is unknown. The existence of *eEF3* orthologs in fungi, algae, some protists, and a few viruses suggests that the last eukaryotic common ancestor (LECA) possessed an *eEF3* gene that was later lost in some taxonomic lineages, including metazoans, land plants, and different protists (Mateyak et al., 2016; Murina et al., 2019). Accordingly, *S. cerevisiae* eEF1A and *eEF2* alone support translation with purified rat liver ribosomes *in vitro*. In contrast, rat liver eEF1A and *eEF2* require yeast *eEF3* to drive translation with yeast ribosomes (Skogerson and Engelhardt, 1977), suggesting that mammalian ribosomes evolved to catalyze protein synthesis in the absence of *eEF3* (Mateyak et al., 2016), therefore losing the *eEF3* gene.

Although there is no ATPase associated with translation elongation in mammals or plants, which is driven by the GTPases eEF1A and *eEF2*, some ABC ATPases play key roles in translation. This is the case of ABCE1, which interacts with release factors and drives the recycling step of translation in eukaryotes. In *S. cerevisiae*, GCN20 mediates ribosome-associated eIF2alpha kinase GCN2 during translation elongation under amino acid starvation (Vazquez de Aldana et al., 1995; Marton et al., 1997) and ARB1 controls ribosome biogenesis (Dong et al., 2005). In mammals, ABC50 interacts with eIF2 and associates with the ribosome to promote translation initiation (Tyzack et al., 2000). In bacteria, ABCF proteins bind the ribosome upon exposure to antibiotics. Etta occupies the ribosomal E site to displace drugs from the ribosome, and both VgaA and MsrE reset peptidyl transferase activity in response to antibiotic treatment (Gerovac and Tampé, 2019). Altogether, these phenomena show that ABC ATPases involved in translation are a common theme in life, supporting the notion that LECA might have possessed *eEF3* activity that was lost later in evolution in some lineages.

### Evolutionary diversification of the elongation factors

The existence of *eEF3* in the fungal kingdom and some protists demonstrates the diversity of factors catalyzing elongation in eukaryotes. Genome-wide studies have revealed that many unicellular lineages lack eEF1A and instead possess a related factor termed the elongation factor-like (EFL) protein, with the residues critical for eEF1A function conserved. *EFL* genes exist in widespread taxa, including green and red algae, some fungi, diatoms, euglenozoans, dinoflagellates, and other protists (Keeling and Inagaki, 2004; Noble et al., 2007; Kamikawa et al., 2008, 2010, 2011; Cocquyt et al., 2009; Gile et al., 2009a,b; Sakaguchi et al., 2009; Atkinson et al., 2014; Mikhailov et al., 2014). Interestingly, *eEF1A* and *EFL* genes display mutually exclusive distributions across taxa. Thus, eEF1A is not universally present in eukaryotes, and it is assumed that eEF1A and EFL are functionally equivalent (Keeling and Inagaki, 2004; Noble et al., 2007; Kamikawa et al., 2008, 2010, 2011; Cocquyt et al., 2009; Gile et al., 2009a,b; Sakaguchi et al., 2009; Mikhailov et al., 2014). Moreover, Murina et al. showed that the taxonomic distribution of *eEF3* across protists is similar to that of *EFL*, i.e., it is present in *EFL*-possessing lineages and is absent in *eEF1A*-containing species (Murina et al., 2019).

Selenium is a trace element present in selenoproteins in the form of the amino acid selenocysteine (Sec). Most selenoproteins are enzymes containing Sec at the active site. Sec is encoded by the stop codon UGA, which is recoded and incorporated into polypeptides by the action of the Sec-specific elongation factor (eEFSec) and a specific tRNA (tRNA-Sec) as part of the Sec machinery (Labunskyy et al., 2014). Selenoproteins and the Sec machinery are essential for vertebrates and are commonly found in most algae (except red algae; Mariotti et al., 2015; Castellano, 2019; Liang et al., 2019; Jiang et al., 2020) but are missing in most insects and land plants. Selenoproteins are highly scattered throughout protists, which include many lineages lacking selenoproteins (Mariotti et al., 2015; Castellano, 2019). Sec was also lost at the root of Dikarya, which comprises the fungal *phyla* Ascomycota and Basidiomycota (Castellano, 2019; Mariotti et al., 2019). The Sec machinery genes were identified in nine fungal species belonging to three early-branching fungal groups, namely Chytridiomycota, Zoopagomycota, and Mucoromycota (Castellano, 2019; Mariotti et al., 2019).

Overall, the studies on *eEF3*, eEF1A, EFL, and eEFSec have unveiled a significant diversification of the translation elongation factors across eukaryotes.

## Conclusion

The investigation of *eEF3* across species within the fungal kingdom is crucial, not only because it shows variability in the translational process but also because of its potential pharmaceutical and genetic research implications. Discoveries in this area not only contribute to the understanding of fundamental factors necessary for translation but also illuminate the processes by which such knowledge can be harnessed for the development of more effective products and technologies. Given that a significant portion of existing knowledge has only been obtained from *S. cerevisiae*, our study beyond this model organism revealed that *eEF3* is essential in pathogenic fungi. This discovery holds promise for a breakthrough in antifungal research as this protein, absent in mammals and plants, could serve as a remarkably specific target.

## Data Availability

The datasets presented in this study can be found in online repositories. The names of the repository/repositories and accession number(s) can be found in the article/Supplementary material.
