# Supplementary material for: The gene YEF3 function encoding translation elongation factor eEF3 is partially conserved across fungi
Source: Front Microbiol. 2024 Aug 23;15:1438900. doi: 10.3389/fmicb.2024.1438900 (PMC11378755; doi:10.3389/fmicb.2024.1438900)
Supplement: Supplementary file 1 [file Table_1.DOCX]

**Supplementary Material of:**

**The gene *YEF3* function encoding translation elongation factor eEF3 is partially conserved across Fungi**

Giovanna Maldonado, Alejandra García, Saturnino Herrero, Irene Castaño, Michael Altmann, Reinhard Fischer, and Greco Hernández

**Supplementary Table S1.** Information of optimized genes

| **Specie** | **Original sequence** | **Optimized sequence** | **Flanking sites** |
| --- | --- | --- | --- |
| *G. prolifera* | ATGCCTCCTAAAAAGAGCAACGCTCGAAACGCCAGCCTCCTGAAGGAAGCTGTCACCAACGCCGGTCGTTCCGACACAGCCTCAACAGCACGGGAAGGCTCAATCGCCGCAACGTTTGTTTCCGACTCGGACTCCTCTACCACCGCCTCTGGACCTGTTGACGAGAAGGTCGCCGCTGTTGCGGAACTCATTACCACACTCACCACGAATGCCGCATCGGACAAGCGTCAATCAGCCGCCGACGACATTGCGAAGTTCGTCGAAGCTGAAGGAGTCGCGCAAATGGTCAAGACTAAGATCCTTCACGGGATCGCTACGGGACTCCACAACACCAAACAACCCGTCGCGCGGGAGGGTGCCATCACGGCTATCGAAACTCTAATTAAGAGCCCCGTTTCGAAGCAAATTGAGCCCTATATGATCACGTTCATTCCGGTACTTCTTGAGCGCTTGTCTGACAAGGCCAAGACTGTCTGCTTGGCGGCGGACTCTGCTTTGAAGGCATTGATTACCAGGCTCACTCCTTACGCCACGAAGCAGGTGCTTCCTCTCCTCCTTGCTGGTATCGAATACAGCCAAAAATGGCAGACCAAGGTTGGAGCCTTAGAGCTGATGCAGAGCCTTTCCAAGACCGCACCTCGCCAAATGACAACGGCGGTGCCGGATCTTGTGCCCCCACTTTCCGAAGTGATGCACGACACCAAAGCGGAAGCAAAGAATGCGGGCCGCCAGACGATGGAGGCCATTTGCGAGCTTATCAACAACAAGGATATTGAAAAATTCATTCCCGCTCTTATCGACACTATCGCAAACCCGGAGAAAGTTCCCGACACTGTTCACCTGCTCGGTGCGACAACATTTGTGTCTGAAGTCCTTCCCCCAACCCTCGCAATCATGGTTCCCCTCTTGTCGCGAGGGCTGAACGAACGTCAGACGGCTATCAAGCGCAAAGCTGCGGTTATTATCATCAACATGTGTAAGCTTGTCGAGAAGCCGCAGATCATTGCGCCTTTCCTCCCGCGCCTGCTGCCAACTCTCGAGAAGATCCAAGACGATGTTGCCGATCCCGAGTGTCGCCAAGTCTGTCAAAATGCAACCAAGATCCTCACCAAGGTCGGAATTCCCGCCCCTGGCACCGTCATGGATGACAGCTACGAATTCAAGAAGTTGGACGATAAGGCTTTGGCATACCTGAAGAAAGCCATTGGTCACCAGCAAATCACAGAAATTCCAGAGGCCTATCGTCCGGTCCTAGAGTACCTGGAACACATTGCAGCTGCCATGGTTGCCGATGGAAACATGGACAATGACGATTGGAACGGTTCCATCATTCCCACCCTGAAAGCTTTTATTCCTACCGAAGGCACAGTAACAAACGCTGTCAAGGAGTTCCGGGAGCTTTCCTTCGGTTCAAATGTCAAGGAGGCTGTCGCTGAAGAGGAGGAAGATGACGCGGAAGAACTTTGCAACTGCGAGTTTTCTCTCGCGTACGGTGCGCGTGTCCTTCTGAACCGCACCCACCTGATTCTCAAGAGGGGTCATCGATATGGGCTTACTGGTGCCAACGGGTCCGGAAAGTCCACCTTGATGCGCGCCATTGCCAACGGACAGGTCGAAGGTTTCCCTCCTGCAGACCAGCTCAAGACTGTCGCTGTAGAACACGACCTGGACGGTGCTCACGAGCACGACAACAAGGAAGTGCAAGCCTTCGTTCTCGAGGACCCGGTCTACGCCCACTTGCCCAAAGAGACTGTCGTCAATATGCTCGAATCTGTCGGCTTCAAAGGCCCCATGGCCACCAGACCAGTCGGTCAACTATCTGGGGGTTGGAGAATGAAGCTCGCCCTCGCCAGGGCTATGCTCCACAATGCCGACATTCTTCTTTTGGACGAACCGACAAACCACTTGGATGTAATCAACGTGGCGTGGTTGCAAGACTACCTCATCGGTTTGAAAACTGTAACGTCGATCATCGTGTCGCACGACTCCAGCTTCTTGGACATAGTGTGCTCTGACATCATCCATCTGGACAACTTCAAGCTCAAGAGGTACCGCGGTAACCTCTCCAAGTTTGTGGAGGCAGTACCCGAGGCCAAAGCTTACTACGAGCTCGGAGCTGCCCAACAAACGTTCAGATTCCCTGAACCTGGTATGCTTGAAGGAGTCAAGACCAAGGAAAGGGCCATCCTCAAAGTCCAGAACGCCGTGTTCCAGTACCCTGGAACGGACAGGAGACAGCTGAATGGCGTGACCTTCCAGTGCTCGCTTGGGTCCCGCGTTGCTGTCGTTGGACCTAATGGTGCCGGCAAGTCCACCCTCATTAAGCTACTTTGCGGTGTCATTGAGCCCGACAACGGTGTTGTCTGGCGCCACCCCAACCTTCGTATTGCCTATGTCGCCCAACATGCCTTCGAGCATATCGAGAAGCACACCAACCTTACTCCAAACCAGTACATCCAATGGCGTTACCAAACCGGTGAAGATCGTGAGGAGATGGAAAAGGCTGCCCGCCAGATCTCCGCAGAAGAGGAGGCTGCGATGAAAAAGGTTCAAGTTATTTCTGGGGAGAAGAAGGTTGTGGACTCAGTCATTGGCAGACGCAAGCTGAAAAACTCGTACGAGTATGAGGTCTCCTGGGTCGGCAAGCTTTCAAATGAGAACTCTTGGCTCCCCCGAGACACTCTTATCGAAATGGGCTTCCTTAAGAAGGTTCAAGAAATTGACCAGGCAGAGGCAGCCAGACAAGGTCTTGCTCGGCCTCTGACCCAAAAGGAAATCGAGAAGCACCTTTCCGACGTCGGTATCGATTCTGAAATCGGCACGCACTCTCACATTCGCGGTCTTTCTGGAGGTCAGAAGGTTAAGGTAGTAATCGCTGGTGCCATGTGGCAACGTCCTCACCTTCTCGTCCTCGACGAACCAACCAACTTCTTGGACCGAGACAGTTTGGGTGCCCTGAAGACTGCAATTGACGCTTACGGCGGTGGTGTGATCATGGTCACCCACAGCAGGGAGTTCTCCGAAGCCATCTGCAAGGAAGTGTGGAAAGTGGACAACGGGGAGCTGACTCCAACTGGTCACAACTGGGTTTCTGGCCAGGGAAGCGGACCCAGGCTTGAAGATAAGAACAAGGACGAAGAAGTCTTCGATGCGTTCGGCAACAAGATCGACGTCGCCAAGCAGAAGAGCAAACTCTCCGGTAAGGACCTTCGGAAGAAGCGAAAAGAGCGCGAAGCCCGCCGCAAGCGTGGTGAGGAAGTGTCTGATGACGACGAGTACCCATACGATGTTCCAGATTACGCTTAA | ATGCCACCAAAGAAATCTAACGCTAGAAACGCATCATTGTTGAAGGAAGCAGTTACAAATGCTGGTAGATCTGATACTGCTTCAACAGCAAGAGAAGGTTCTATTGCTGCAACTTTTGTTTCTGATTCAGATTCTTCAACTACAGCTTCAGGTCCAGTTGATGAAAAAGTTGCTGCAGTTGCAGAATTGATCACTACATTGACTACAAATGCTGCATCTGATAAAAGACAATCAGCTGCAGATGATATTGCTAAATTTGTTGAAGCAGAAGGTGTTGCTCAAATGGTTAAGACAAAGATCTTGCATGGTATCGCAACTGGTTTGCATAACACAAAGCAACCAGTTGCTAGAGAAGGTGCTATCACTGCAATCGAAACATTGATTAAATCTCCAGTTTCAAAGCAAATCGAACCATACATGATCACTTTTATTCCAGTTTTGTTGGAAAGATTGTCTGATAAGGCTAAGACTGTTTGTTTAGCTGCAGATTCAGCTTTGAAGGCATTGATCACTAGATTGACACCATACGCAACTAAGCAAGTTTTGCCATTGTTATTGGCTGGTATCGAATACTCTCAAAAGTGGCAAACAAAAGTTGGTGCTTTGGAATTGATGCAATCTTTGTCAAAGACTGCACCAAGACAAATGACTACAGCTGTTCCAGATTTGGTTCCACCATTATCAGAAGTTATGCATGATACTAAAGCAGAAGCTAAAAATGCAGGTAGACAAACAATGGAAGCTATTTGTGAATTGATTAATAATAAGGATATCGAAAAGTTTATTCCAGCATTGATCGATACTATCGCTAACCCAGAAAAAGTTCCAGATACAGTTCATTTGTTGGGTGCTACTACATTTGTTTCTGAAGTTTTGCCACCAACATTAGCAATTATGGTTCCATTATTGTCAAGAGGTTTGAACGAAAGACAAACTGCTATTAAAAGAAAGGCTGCAGTTATTATTATTAACATGTGTAAGTTGGTTGAAAAGCCACAAATCATCGCTCCATTTTTGCCAAGATTGTTGCCAACTTTGGAAAAGATTCAAGATGATGTTGCAGATCCAGAATGTAGACAAGTTTGTCAAAACGCTACTAAGATCTTGACAAAAGTTGGTATTCCAGCACCAGGTACAGTTATGGATGATTCTTACGAATTCAAGAAATTGGATGATAAGGCATTGGCTTATTTGAAGAAAGCTATTGGTCATCAACAAATCACTGAAATCCCAGAAGCATACAGACCAGTTTTGGAATACTTAGAACATATTGCTGCAGCTATGGTTGCTGATGGTAATATGGATAACGATGATTGGAACGGTTCTATCATCCCAACTTTGAAGGCTTTTATTCCAACAGAAGGTACTGTTACAAATGCAGTTAAGGAATTCAGAGAATTGTCTTTCGGTTCAAATGTTAAAGAAGCAGTTGCTGAAGAAGAAGAAGATGATGCTGAAGAATTGTGTAACTGTGAATTTTCATTGGCTTACGGTGCAAGAGTTTTGTTGAACAGAACACATTTGATCTTGAAAAGAGGTCATAGATACGGTTTGACTGGTGCTAATGGTTCTGGTAAATCAACATTGATGAGAGCAATCGCTAATGGTCAAGTTGAAGGTTTTCCACCAGCAGATCAATTGAAAACTGTTGCTGTTGAACATGATTTGGATGGTGCTCATGAACATGATAATAAGGAAGTTCAAGCATTCGTTTTGGAAGATCCAGTTTATGCTCATTTGCCAAAGGAAACAGTTGTTAACATGTTGGAATCTGTTGGTTTTAAAGGTCCAATGGCTACTAGACCAGTTGGTCAATTGTCAGGTGGTTGGAGAATGAAATTGGCATTAGCTAGAGCAATGTTGCATAACGCTGATATCTTGTTGTTGGATGAACCAACTAACCATTTGGATGTTATTAATGTTGCATGGTTGCAAGATTATTTGATTGGTTTAAAAACTGTTACATCTATTATTGTTTCACATGATTCTTCATTTTTAGATATTGTTTGTTCTGATATCATCCATTTGGATAACTTCAAGTTGAAAAGATACCGTGGTAATTTGTCAAAGTTCGTTGAAGCTGTTCCAGAAGCTAAAGCATATTACGAATTAGGTGCAGCTCAACAAACTTTTAGATTTCCAGAACCAGGTATGTTGGAAGGTGTTAAGACTAAGGAAAGAGCAATCTTGAAAGTTCAAAATGCTGTTTTTCAATATCCAGGTACTGATAGAAGACAATTGAACGGTGTTACATTCCAATGTTCTTTAGGTTCAAGAGTTGCAGTTGTTGGTCCAAATGGTGCTGGTAAATCTACATTGATTAAATTGTTGTGTGGTGTTATTGAACCAGATAATGGTGTTGTTTGGAGACATCCAAATTTGAGAATTGCTTACGTTGCACAACATGCTTTCGAACATATCGAAAAGCATACTAATTTGACACCAAACCAATACATCCAATGGAGATACCAAACTGGTGAAGATAGAGAAGAAATGGAAAAAGCAGCTAGACAAATTTCTGCTGAAGAAGAAGCAGCTATGAAGAAAGTTCAAGTTATTTCTGGTGAAAAGAAAGTTGTTGATTCAGTTATTGGTAGAAGAAAGTTGAAAAATTCTTACGAATACGAAGTTTCATGGGTTGGTAAATTGTCTAACGAAAACTCATGGTTGCCAAGAGATACTTTGATCGAAATGGGTTTCTTGAAGAAAGTTCAAGAAATCGATCAAGCAGAAGCAGCTAGACAAGGTTTGGCTAGACCATTGACACAAAAGGAAATCGAAAAGCATTTGTCTGATGTTGGTATCGATTCAGAAATCGGTACTCATTCTCATATCAGAGGTTTATCAGGTGGTCAAAAAGTTAAAGTTGTTATTGCTGGTGCAATGTGGCAAAGACCACATTTGTTGGTTTTGGATGAACCAACTAATTTCTTGGATAGAGATTCTTTGGGTGCTTTAAAAACAGCAATTGATGCTTATGGTGGTGGTGTTATTATGGTTACTCATTCTAGAGAATTTTCAGAAGCTATCTGTAAGGAAGTTTGGAAGGTTGATAATGGTGAATTGACTCCAACAGGTCATAATTGGGTTTCTGGTCAAGGTTCAGGTCCAAGATTGGAAGATAAGAATAAGGATGAAGAAGTTTTCGATGCATTCGGTAATAAGATCGATGTTGCTAAGCAAAAATCTAAGTTGTCTGGTAAAGATTTGAGAAAGAAAAGAAAGGAAAGAGAAGCTAGAAGAAAGAGAGGTGAAGAAGTTTCTGATGATGATGAATATCCATACGATGTTCCAGATTACGCATAA | *BamHI/SmaI* |
| *A. nidulans* | ATGGCTTTCAGAGTACCTGACCTAATCCCAGCTTTATCCGCTAACATCTGGGACACCAGAGATCCAGTCAAAGACAGAATCCAAGGTACTCCTTACAAGCCTTACCAACCAGCTAAGGGTAAAAAGCCAGAACAAAAGGAACAACCAGAAAGAACCGGTACCTTGGAGATAGTCTGTGGTTTGATTTCCAACAAGGATATTGAAAAATTCATCCCAGCTTTGGTCAAGTGTATCGCGTTCCCAGATCGAGTTCCAGAAACTATTCACCTGTTAGGTGCTACCACTTTCGTCTCTGAAGTCACAGGTCCTACCTTGGCCATTATGACCCCACTATTGGAACGTGGTTTGAAAGCTGAACAAGCTACCCCAATAAAGCGTAAGGCCGCTGTTATCGTTGATAACATGTGTAAGCTCGTTGAAGATCCTCAAATTGTTGCTCCATTCTTGCCATTGTTGCTACCACAAATCTTGAAGCTTTCAGGTGAATCTCCAGATCCAAAGGAAAACGGTTTGGCAGACCCAGAAGCTAGAGGTAAGTGCAAGCAAGCTTTAGATACTTTGACTCGTGTTGGTAACGTCGTCGATGGTAAGATTCCAGAAATTTCAACCGCCGGTGATATTTCCACTGTTTCCGCCATTTTGAAGGACATTCTAGCCGCCAAGTTCAAATCTCAAGCTGAAAAAGCTGAAGCTGTCATTAACTACGTTGCTGCTATTGCTGGTCAATTAGTAGATGAAAAGATTGCTGAATCCGCTGACTGGACCAGAAACGTGTTGCCATACATCGCTGCCATTGTTGGTGAAGCTGATGCCCCAGCTATCGCTGAAACCTTGCGTAAAAGAGCTTCTCCAGACGCCGCTGCTGCTGACGCTGTCGAATCTGACGAAGAGGAAGGTGAAGACTTGTGTAACTGTACCTTTTCTCTAGCTTACGGTGCCAAGATCTTATTGAACCAAACCAGTTTGAGGTTGAAGAGAGGTCAAAGATACGGTTTGTTAGGTCCAAACGGTTCTGGTAAGACCACATTGATGAGAGCCATCAACAACGAACAATTGGAAGGTTTCCCAAAGAAGGACGAAGTCAAAACCGTTTACGTCGAACACGACTTGGACTCCGCAGACACTGAACAAACTGTAATTGGTTGGACCATGAAGAAGTTGAGAGAAGTTGGTTTGGACCCAGTTCAATCTGAAGTTGAATCCAAGTTGGAAGAATTTGGTTTCCTCAGAGAACAGTTTGAAGGACCAATCACTGCTTTGTCTGGTGGTTGGAAGATGAAATTGGCCCTATGTAGAGCTGTTTTCGAAGACCCAGACATCTTGTTGTTGGATGAACCAACCAACCATTTGGATGTCAAGAACGTTGCTTGGTTGGAAAATTATCTAAGAAACTCTCCATGCACCTCTATTATTGTCTCCCACGATTCTAAGTTCTTGGACAATGTTTTGCAACATGTTATCCATTACGAAAGATTCAAGTTGAAGAGATACAGAGGTACCTTAAGCGAATTTGTTAAGAGAGTTCCATCTGCTAGATCTTACTACGAATTGGGTGCTTCGGATTTGGAATTTAAGTTCCCAGAACCAGGTTTCTTGGAAGGTGTTAAGACCAAGGCAAAGGCTATCATCAGAGTCTCTAACATGTACTTCAGATACCCAGGTGTCGACCGTGACCAACTGCAAGATATCACTTTCCAAGTCTCCTTGGGTTCTAGAATCGCCGTTATTGGTCCAAACGGTGCTGGTAAGTCTACGCTCGTTGACGTTTTGACTGGTGAAAAGATCGCCACCAGAGGTGAAGTGTACCACCACGAAAACATCAGAATTGCTTATATCAAACAACACGCATTCGCTCACATCGACAACCACTTGGACAAGACTCCTTCTGAATACATTCAATGGAGATTCCAAACTGGTGAAGACAGAGAAACCATGGACAGAGCCAACAAGCAAGTTACTGAAGAAGATGAAAAGGCTGAATTAAAGATCTACAAGATCGAAGGTACTCCTCGTCGTGTTGTCGGTGTCCACGCTAGAAGAAAGTTTAAGAACACTTACGAATATGAATGTAGTTTCTTGTTGGGAGAAAACATTGGTATGAAGTCCGAAAAGTGGACTCCAATGTCTACTACCGACAACGCTTGGATCCCAAGAAATGAAATCATGGAATCCCATAGCAAGAAGGTCGCTGAAATTGACGCTCAAGAAGCCTTGAAGTCAGGTAACATTAGACCATTGATCAGAAAGGAAATTGAAGACCACTGTGCTCAATTCGGTCTGGATGCTGAATTGGTTTCTCACTCCCAAATGAGAGGCTTGTCCGGTGGTCAACGCGTTAAAGTTGTTTTAGCCGCTTGTTCCTGGCAAAGACCACACGTTATTGTCTTGGATGAACCAACTAACTACTTGGACAGAGACTCTTTGGGTGCTTTGTCTAAGGCTTTAAAGGCTTTCGAAGGTGGTGTCATCATCATAACTCACTCTGCCGAATTTACTCAAAACTTGACCGAAGAAGTCTGGGCTGTCTTGGACGGTAAGATGACTCCAAGCGGTCACAACTGGGTTACCGGTCAAGGTTCTGGCCCAAGATTGGAAGAAAAGGAAGGTCCAGATGAGATTGTCGATGCTATGGGTAACAAGATCAAGGTCGAAAAGAAGGTTAAGTTGTCCTCTGGTGACAAACGGAAGCAAAGAAAGGAAAGATTAGCCAGAAAGAAAAGAGGAGAAGATGTTGATGATGACGAAGATTTTTGA | ATGGCTTTCAGGGTCCCAGATTTGATTCCAGCTTTGAGTGCTAACATCTGGGACACTAGAGACCCAGTTAAGGACAGAATCCAAGGTACCCCATACAAGCCTTACCAACCAGCTAAGGGTAAAAAGCCGGAACAAAAGGAACAACCAGAAAGAACTGGTACTTTGGAAATTGTCTGTGGTCTAATCTCTAACAAAGACATTGAAAAGTTCATCCCAGCCTTGGTCAAGTGTATCGCCTTCCCAGACCGTGTCCCTGAAACTATCCACTTGTTGGGTGCTACCACTTTCGTCTCCGAAGTTACAGGTCCAACCTTGGCTATCATGACTCCTCTGTTGGAAAGAGGCTTGAAGGCCGAACAAGCTACTCCAATCAAGAGAAAAGCGGCCGTTATAGTCGATAACATGTGTAAGTTGGTTGAAGATCCCCAAATTGTTGCTCCATTCTTGCCATTGTTATTGCCACAAATCTTGAAGTTGAGCGGTGAGTCTCCTGATCCAAAGGAAAACGGTTTAGCTGATCCAGAAGCTAGAGGTAAGTGCAAGCAAGCCCTAGATACCTTGACCCGTGTTGGTAACGTTGTCGATGGTAAGATCCCAGAAATTTCCACTGCTGGTGACATCTCTACAGTTTCCGCCATCTTGAAGGACATCTTGGCCGCCAAGTTTAAGTCCCAGGCTGAAAAAGCTGAAGCTGTTATCAACTACGTTGCCGCCATTGCTGGTCAATTAGTTGATGAAAAGATTGCTGAATCTGCTGACTGGACCAGAAACGTACTACCATATATCGCTGCCATCGTTGGTGAAGCCGACGCTCCCGCTATCGCCGAAACTTTGAGAAAGAGAGCTTCGCCAGATGCTGCTGCTGCTGATGCAGTCGAATCCGATGAAGAAGAAGGTGAAGATTTATGTAACTGTACCTTCTCTCTGGCTTACGGTGCTAAGATTTTGTTGAACCAAACCTCCTTGAGATTGAAGAGAGGCCAAAGATACGGTTTGTTGGGTCCAAACGGTTCCGGTAAGACCACCTTGATGAGAGCTATCAACAACGAACAATTGGAAGGTTTTCCAAAGAAGGATGAAGTTAAGACTGTTTACGTCGAACACGACTTAGACTCCGCTGACACTGAACAAACTGTTATTGGTTGGACCATGAAGAAATTGCGTGAAGTTGGTTTAGACCCAGTCCAATCTGAAGTGGAAAGTAAGCTCGAAGAATTTGGTTTCTTGAGAGAACAATTTGAAGGTCCAATTACCGCTTTGTCTGGTGGTTGGAAGATGAAATTAGCCTTGTGTCGTGCTGTATTCGAAGACCCAGACATTTTGCTGTTGGACGAACCAACCAACCACTTGGATGTCAAGAACGTTGCTTGGTTAGAAAACTATTTGCGAAACTCTCCATGCACTTCCATCATTGTCTCTCACGACTCCAAGTTTTTGGATAACGTCTTGCAGCATGTCATCCATTACGAAAGATTCAAGTTAAAAAGATACAGAGGTACTTTGTCTGAATTTGTTAAGAGAGTTCCATCCGCCAGATCTTACTACGAACTTGGTGCTTCTGATTTGGAATTTAAGTTCCCAGAACCAGGTTTCCTAGAAGGTGTCAAGACCAAGGCTAAAGCTATCATCAGAGTTTCCAACATGTACTTCAGATACCCAGGTGTCGACAGGGACCAATTGCAAGATATTACCTTCCAAGTTTCTTTGGGTTCTAGAATTGCTGTCATCGGTCCAAACGGTGCCGGTAAGTCTACTCTTGTCGATGTTTTGACTGGGGAGAAGATCGCTACCAGAGGTGAAGTCTACCACCACGAAAACATTCGTATTGCTTACATCAAGCAACACGCTTTCGCTCACATTGACAATCATCTGGATAAGACTCCATCTGAATACATTCAATGGAGATTCCAAACCGGTGAAGACAGAGAAACCATGGACAGAGCTAATAAGCAAGTTACTGAAGAAGACGAAAAAGCTGAATTGAAGATCTACAAGATCGAAGGTACCCCAAGAAGAGTTGTTGGTGTCCACGCTAGAAGAAAGTTCAAGAACACTTACGAATATGAATGTTCTTTCTTATTAGGTGAAAACATTGGTATGAAGTCTGAAAAATGGACTCCAATGTCTACTACCGACAACGCTTGGATTCCAAGAAATGAAATCATGGAATCCCATTCCAAGAAGGTTGCTGAAATTGATGCCCAAGAGGCTTTGAAATCAGGTAACATCAGACCATTGATCCGTAAGGAGATCGAAGACCACTGTGCTCAATTCGGTTTGGATGCTGAATTGGTTTCTCACTCTCAAATGAGAGGTTTGTCTGGCGGTCAAAGAGTCAAGGTCGTTTTGGCTGCTTGTTCCTGGCAAAGACCACACGTTATTGTTTTGGACGAACCAACTAACTACTTGGACAGAGATTCTTTGGGTGCTCTGTCTAAGGCTTTGAAGGCTTTCGAAGGTGGTGTTATTATTATCACTCACTCCGCCGAATTTACTCAAAACTTGACCGAAGAAGTCTGGGCTGTCTTGGACGGTAAGATGACCCCATCTGGTCACAACTGGGTTACCGGCCAAGGTTCTGGTCCAAGACTCGAAGAAAAGGAAGGTCCAGATGAAATCGTTGACGCTATGGGTAACAAAATTAAGGTCGAAAAGAAGGTTAAGTTGTCCTCTGGTGACAAGAGAAAGCAAAGAAAGGAAAGATTGGCTCGGAAGAAGAGAGGTGAAGACGTTGACGATGACGAAGATTTCTACCCATACGATGTTCCAGATTACGCTTGA | *BamHI/SmaI* |

**Supplementary Table S2**. Primers used for the phenotypic rescue assays.

| **Cloning fragment** | **Forward primer***  **5’->3’** | **Reverse primer***  **5’->3’** | **Flanking sites** |
| --- | --- | --- | --- |
| *eEF3* ORF *S.cerevisiae* | *GGATCC*ATGTCTGATTCCCAGCAATCCATTA | *CCCGGG*TTA**AGCGTAATCTGGAACATCGTATGGGTA**GAATTCTTCGTCAGAAGAAACGTAAG | *BamHI/SmaI* |
| *eEF3* ORF *C.glabrata* | *GGATCC*ATGACTGACTCTGACCAATCTCTTA | *CCCGGG*TTA**AGCGTAATCTGGAACATCGTATGGGTA**GAAATCTTCGTCATCGGAAGAAACG | *BamHI/SmaI* |
| *eEF3* ORF *U.maydis* | *GGATCC*ATGGCTCCCGCTCCTTCCGCTGCTG | *GAATTC*TTA**AGCGTAATCTGGAACATCGTATGGGTA**AAGGTCCTCGAGCTCATCAGCCGAG | *BamHI/EcoRI* |
| *eEF3 ORF Z. rouxii* | *GGATCC*ATGTCTGACTCGCAACAATCCGTCG | *CCCGGGTTA***AGCGTAATCTGGAACATCGTATGGGTA**GAAGTCTGCATCGTCATCGGACACA | *BamHI/SmaI* |

*Sequence in italics and underlined refers to the incorporated restriction flanking sites. Sequence in bold refers to HA tag:

TAC CCA TAC GAT GTT CCA GAT TAC GCT (YPYDVPDYA).

**Supplementary Table S3.** eEF3-HA protein size from species used in this study.

| **Specie** | **eEF3-HA protein expected size (kDa)** |
| --- | --- |
| *S. cerevisiae* | 116 |
| *C. glabrata* | 116 |
| *U. maydis* | 118 |
| *Z. rouxii* | 115 |
| *G. prolifera* | 121 |
| *A. nidulans* | 103 |

**Supplementary Table S4**. Primers used for eEF3 deletion in *C. glabrata* and *A. nidulans.*

| **Cloning fragment** | **Forward primer***  **5’->3’** | **Reverse primer***  **5’->3’** | **Flanking sites** |
| --- | --- | --- | --- |
| *NAT^R^* ORF | *GAATTC*ATGGGTACCACTCTTGACG | *GGATCC*TTAGGGGCAGGGCATGCTCATGTAG | *EcoRI/BamHI* |
| LB flanking region *eEF3* *C.glabrata* | *CTCGAG*TTAAAGGTCCACATAGACGTACC | *GAATTC*TTGTCAGATGTCTCTCTTTTC | *XhoI/EcoRI* |
| RB flanking region *eEF3* *C.glabrata* | *GGATCC*GTGAATCTCATCTTTTAATC | *CTCGAG*AAATATCATCGACCCATATAC | *BamHI/XhoI* |
| PCR strategy for cassette insertion | TGGCGGACCCATGGCTGAACACGTT | *GGATCC*TTAGGGGCAGGGCATGCTCATGTAG |  |
| LB flanking region *eEF3* *A. nidulans* | GAAGAGCATTGTTTGAGGCGGACGGAAGCAATATCAACGGCAG (P1) | CCAGAAGTCGACAAATTACATACCGTTC (P3) |  |
| RB flanking region *eEF3* *A. nidulans* | ATCAGTGCCTCCTCTCAGACAGGGCGAGGATGTTGATGATGATGAGG (P5) | AGAACAACAATAGCGCCCTCG (P8) |  |
| *pyrG* *A. fumigatus* | CGCCTCAAACAATGCTCTTCAC (pyrG-F) | CTGTCTGAGAGGAGGCACTGATG (pyrG-R) |  |
| PCR-fusion nested primers | CAAGTTATATGGATTTAGCTGCGCCAC (P2) | TGGCTCGTTTGGCTGGTTTG (P7) |  |
| PCR strategy for cassette insertion | AGCTTTGCCAGAAACGCCAAG | AGAACAACAATAGCGCCCTCG (P8) |  |

*Sequence in italics and underlined refers to flanking sites.
